# Serially Checked Spherical Aberration Can Evaluate the Anti-Myopia Effect of Orthokeratology Lens in Children

**DOI:** 10.3390/jpm12101686

**Published:** 2022-10-10

**Authors:** In-Kyun Hahn, Donghan Lee, Dong-Ho Lee, Hun Lee, Hungwon Tchah, Jae-Yong Kim

**Affiliations:** 1 Department of Ophthalmology, University of Ulsan College of Medicine, Asan Medical Center, 88, Olympic-Ro 43-Gil, Songpa-gu, Seoul 05505, Korea; 2 University of Ulsan College of Medicine, 88, Olympic-Ro 43-Gil, Songpa-gu, Seoul 05505, Korea; 3 Bitsarang Eye Clinic, Prince Building, 492, Nohae-ro, Nowon-gu, Seoul 01751, Korea

**Keywords:** myopia, orthokeratology lens, higher-order aberration, spherical aberration, aberrometer

## Abstract

We aimed to investigate the changes in higher-order aberrations (HOAs) after wearing orthokeratology (OK) lenses in myopic patients. The study included 15 eyes from ten myopic patients, whose refractive error was myopia less than −4.5 diopters (D) and astigmatism less than 1.5 D. Uncorrected visual acuity (UCVA) and best-corrected visual acuity (BCVA) were measured, and Zywave^®^ aberrometry was performed at baseline and 1, 3, and 6 months following OK lens wear. The mean age was 11.5 years (range: 9–15 years). There was a significant improvement in UCVA (*p* ≤ 0.001) and a decrease in the spherical equivalent measured with auto-refraction at 6 months (*p* ≤ 0.001). Total HOAs significantly increased after OK lens wear (*p* ≤ 0.001), with spherical aberration increasing approximately 3.9-fold (*p* = 0.05). Spherical aberration demonstrated statistically significant positive correlations with the change in spherical equivalent at 3 and 6 months (*p* = 0.007 and 0.003, respectively). After wearing properly prescribed OK lens, all subjects had significantly improved UCVA and decreased myopic spherical equivalent, with increased total HOAs and positive spherical aberration at 1 month, and the changes were maintained at 6 months. Serially checked spherical aberration could evaluate the anti-myopia effect of the orthokeratology lens in children.

## 1. Introduction

The orthokeratology (OK) lens is a contact lens used for correction of refractive errors such as myopia, hyperopia, and astigmatism by temporarily modifying the shape of the cornea [1,2,3,4]. These lenses are typically prescribed for patients who do not want to wear contact lenses during the day. OK lenses are also prescribed to children to prevent the progression of myopia [5,6,7,8,9,10]. When OK lenses induce physical changes in the corneal shape, there is an accompanying change in refractive error, along with the associated higher-order aberrations (HOAs) [11,12,13]. According to Stillitano et al. [13], defocus decreased at 8 days after wearing OK lenses, then stabilized. However, total HOAs significantly increased, with a 7-fold increase in spherical aberration. Lian et al. [12] reported that OK lenses changed mid-peripheral corneal thickness in the vertical and horizontal meridians, in addition to increasing both corneal and ocular HOAs.

In the present study, we attempted to investigate the changes in visual acuity, refraction, keratometric parameters, and HOAs, and to know whether the higher-order aberrations including spherical aberration were correlated with myopic improvement in children after 6 months of OK lens wear.

## 2. Materials and Methods

### 2.1. Subjects

Eleven myopic patients who commenced OK lens wear in the Bitsarang Eye Clinic between December 2014 and March 2015 were enrolled in this retrospective study. All subjects had less than 4.5 diopters (D) of myopia and astigmatism less than 1.5 D, with corrected visual acuity of 20/20. Excluding refractive error, no history of ocular disease, surgery, or trauma were noted among the study participants during medical record reviews. The study was approved by the hospital Ethics Committee and followed the tenets of the Declaration of Helsinki.

### 2.2. Study Measurements

Uncorrected visual acuity (UCVA) and best-corrected visual acuity (BCVA) were measured on the logMAR scale. Keratometric readings (K) and spherical equivalent were obtained with an auto-refractometer (KR-8800, Topcon Corp., Tokyo, Japan). HOAs were measured with the Zywave^®^ aberrometer (Bausch & Lomb, Inc., Rochester, NY, USA), and corneal topography (Orbscan IIz, Bausch & Lomb, Inc.) was also performed on all subjects. All examinations were carried out at the initial visit and repeated at 1, 3, and 6 months following the commencement of OK lens wear.

Automated refractometry (KR-8800) was used to measure curvature (K) of the central 3 mm of the cornea. It acquired three consecutive K measurements automatically, and gave us an average of K for the analysis. For corneal topography, simulated keratometric readings (Sim K) and steep K and flat K of the central cornea with a radius of 5 mm were measured for the analysis. The pupil size was measured by the Zywave^®^ aberrometer. Zernike polynomials were used to quantitatively express the wavefront aberrations measured by the Zywave^®^ aberrometer at 5 mm pupil size [14,15,16].

### 2.3. Data Analysis

Second order aberrations, third and fourth order HOAs, total HOAs (third and fourth order), and total aberrations were calculated for analysis. Defocus (Z200) and primary astigmatism (Z22) of second order aberrations, coma (Z31) and trefoil (Z330) of third order HOAs, and spherical aberration (SA, Z400), secondary astigmatism (Z420+Z421) and quadrafoil (Z440+Z441) of fourth order HOAs were measured in root-mean-square (RMS, µm) units [14,15,16]. RMS is a unit of measurement for aberrations that can be calculated as the square root of the arithmetic mean of the squares of all constant terms in Zernike polynomials [17]. It implies the difference from the ideal plane that has no aberration, the so-called wavefront, with higher values indicating greater optical imperfection [17].

### 2.4. Statistical Analysis

SPSS software (version 18.0 software for Windows; SPSS, Inc., Chicago, IL, USA) was used for statistical analysis. Analysis of variance (ANOVA) and paired Wilcoxon signed-ranks test were performed to compare the changes in clinical parameters before and after the use of OK lenses. As Mauchly’s sphericity test was not satisfied, the Greenhouse–Geisser correction was performed. To investigate measurement differences at 1, 3, and 6 months compared with baseline, tests of contrasts were performed. Correlation analysis with Spearman’s correlation coefficient was performed to test for a relationship between the changes in aberrations and refractive power. *p* values < 0.05 were considered as statistically significant.

## 3. Results

### 3.1. Patients Demographics

Among 22 eyes of 11 subjects that commenced OK lens wear, 15 eyes from ten subjects were included in this study. In five eyes of five subjects, we failed to obtain reliable aberrometry measurements due to small pupils or poor cooperation. One subject did not present for re-examination during the follow-up period. The mean age of the subjects was 11.5 years (range: 9–15 years) and demographic characteristics are listed in Table 1.

The Paragon CRT^®^ Contact Lens (CRT; Corneal Refractive Therapy, Paragon Vision Sciences, Inc., Mesa, AZ, USA) was used in 11 eyes and the LK^®^ CH2 OK Lens (LK; Lucid Korea, Inc., Seoul, Korea) was used in four eyes. OK lens fitting was performed without difficulty and the lenses were well-tolerated in all subjects. A central bull’s eye pattern was confirmed in topographic examination at 1, 3, and 6 months after OK lens wear in all subjects.

### 3.2. Visual Acuity, Refractions, and Topography

Mean UCVA (logMAR) was 0.96 ± 0.12 at baseline and improved to 0.01 ± 0.02 at 6 months (*p* ≤ 0.001). Mean spherical equivalent measured by auto-refractometry was −2.88 ± 0.68 D at baseline and decreased to −0.59 ± 0.36 D at 6 months (*p* ≤ 0.001), showing the greatest decrease at 3 months (*p* ≤ 0.001). Mean K measured by auto-refractometry decreased from 43.49 ± 1.03 D at baseline to 41.92 ± 1.34 D at 6 months (*p* ≤ 0.001), showing the greatest decrease at 3 months (*p* ≤ 0.001; Table 2).

In corneal topography, the steep and flat K of Sim K significantly decreased after OK at all follow-up periods (*p* ≤ 0.001 and *p* ≤ 0.001, respectively). Astigmatism measured by the Sim K also decreased but was not statistically significant (*p* = 0.10). Both steep and flat K of the central 5 mm corneal radius significantly decreased (*p* = 0.007 and *p* ≤ 0.001, respectively), but astigmatism increased significantly at 1 month and was maintained at 6 months (*p* = 0.05). Moreover, the pupil size measured with the aberrometer did not show statistically significant changes at 1, 3, and 6 months (*p* = 0.72; Table 2).

### 3.3. Higher-Order Aberrations

Table 3 shows the aberration values measured at baseline, 1, 3, and 6 months. Total HOAs increased significantly at 1, 3, and 6 months (all *p* ≤ 0.001). Total aberrations showed a significant decrease at all follow-up examinations (all *p* ≤ 0.001) and the greatest decrease was at 3 months. Defocus (Z200) significantly decreased at all follow-ups (all *p* ≤ 0.001). 0° astigmatism (Z220) showed no statistically significant change at 1 month (*p* = 0.35), but showed a significant decrease from the baseline at 3 months (*p* = 0.01) and the decrease was maintained at 6 months (*p* = 0.03). Astigmatism at 45° (Z221) had no statistically significant changes (*p* = 0.16). Vertical coma (Z311) significantly increased in the negative direction at 1 month (*p* ≤ 0.001) but decreased to an insignificant value at 3 months (*p* = 0.78), and the change was maintained at 6 months (*p* = 0.73). Horizontal coma (Z311) and trefoil (Z331) did not change significantly at any follow-up (*p* = 0.35 and *p* = 0.21, respectively). SA (Z400) significantly increased at all follow-ups with the greatest increase at 6 months (*p* = 0.05). Secondary astigmatism (Z420+Z421) had no statistically significant change (*p* = 0.42). Quadrafoil (Z440+Z441) showed a significant change in the negative direction at 1 month (*p* = 0.005), but showed a positive effect change at 3 months (*p* = 0.01), then a negative change again at 6 months (*p* = 0.01), showing a significant change in negative direction overall (*p* = 0.03).

SA (Z400) demonstrated statistically significant positive correlations with the change in spherical equivalent at 3 and 6 months (Spearman correlation = 0.679 and 0.707, respectively; *p* = 0.007 and *p* = 0.003, respectively). Other than SA, the remaining aberrations including total HOAs showed no statistically significant correlations with the correction of refractive error.

## 4. Discussion

Orthokeratology lenses temporarily reshape the cornea to change the refractive power, thereby correcting the refractive error of the eye [2,3,4,5]. It is noteworthy that there is a paucity of reporting regarding the higher-order aberrations in relation to OK lens use by pediatric patients. To date, the present study is the first to quantify and describe the changes in HOAs and corneal astigmatism following OK lens wear only in the pediatric population.

The measurement of wavefront aberrations provides a relative value that indicates the deviation from a perfect wavefront. Total wavefront can be converted by the sums of aberrations with selective weight; the most frequently-used method is that of Zernike polynomials [14]. When an OK lens induces a physical change in the cornea, this affects the corneal HOAs as well as its refractive power [12,13,18]. HOAs as well as intraocular scatter can decrease image quality focused on the retina, and consequently, reduce subjective visual performance [19]. Because contrast sensitivity (CS) turns out to be clinically useful for assessing the subjective visual performance [20], it might be helpful to check the CS to investigate the subjects’ visual performance in the present study. In this study, total HOAs increased from 0.231 ± 0.089 µm (RMS) at baseline to 0.595 ± 0.205 µm at 1 month after the use of an OK lens, then continuously increased to 0.654 ± 0.219 µm at 6 months. In their study, Stillitano et al. [13] reported that total HOAs significantly increased from 0.41 ± 0.12 µm to 1.04 ± 0.32 µm 8 days after wearing OK lenses, and the change was maintained at 1 year follow-up. The increase in total HOAs after OK lens wear has been also described in several other studies [12,18,21]. Stillitano et al. reported an approximate 7-fold increase in SA (Z400) at 8 days after initiation of OK [13]. In our study, SA (Z400) increased from 0.170 ± 0.105 µm at baseline to 0.608 ± 0.197 µm at 1 month, an approximate 3.6-fold increase, with the change being maintained at 6 months. Similar effects have been reported by other researchers; Lian et al. showed a 3.9-fold increase in SA (Z400) after 1 month [12], and Gifford et al. noted an increase in SA (Z400) after 7 days [18]. It has been hypothesized that this change might be caused by OK lens-associated corneal reshaping, an adaptive response in which the central and peripheral cornea flattens, whereas the mid-peripheral cornea steepens. Stillitano et al. reported that defocus (Z200) decreased until 8 days following OK lens wear and then stabilized [13]. Defocus (Z200) also significantly decreased from −4.270 ± 1.427 µm at baseline to −3.113 ± 1.532 µm at 1 month, and was maintained at 6 months in our study.

Considering astigmatism, Lian et al. reported an increase in this lower-order aberration (Z220+Z221), but 0° and 45° astigmatism were not measured separately [12]. In our study, 0° astigmatism (Z220) decreased from 0.401 ± 0.555 µm at baseline to 0.321 ± 0.609 µm at 1 month, then significantly decreased to 0.149 ± 0.616 µm at 3 months, and was maintained at 6 months. There was no statistically significant change in 45° astigmatism (Z221). Similarly, there was no statistically significant change in astigmatism (Z220+Z221) reported from Stillitano et al.’s work [13]. In our study, keratometric astigmatism measured by automated refractometry did not differ significantly before and after OK lens wear, showing a similar result to that measured by aberrometry. This implies that conventional OK lens wear has relatively low capability to correct astigmatism. Moreover, patients with astigmatism greater than 1.5 D are recommended to use toric OK lenses rather than conventional OK lens.

Horizontal coma (Z310) showed an effect change in the positive direction, but this was not statistically significant. Conversely, vertical coma (Z311) had a significant change in the negative direction from −0.002 ± 0.153 µm at baseline to −0.463 ± 0.351 µm at 1 month. However, this changed to −0.086 ± 0.244 µm at 3 months, showing no significant difference from the baseline, and was maintained at 6 months. Gifford et al. [18] reported an increase in coma (Z310+Z311) at 7 days and Lian et al. [11] also reported an increase in coma (Z310+Z311) at 1 month, but there was no separate analysis investigating difference between horizontal and vertical coma.

Of the other HOAs, Stillitano et al. noted that trefoil (Z331) changed in the positive direction at 30 days and then stabilized, and quadrafoil (Z440+Z441) changed in the negative direction at 30 days but re-calibrated to a similar value as the baseline at 3 months [13]. In our study, trefoil (Z331) decreased from 0.095 ± 0.124 µm at baseline to 0.055 ± 0.100 µm at 1 month and was then maintained, but this was not a statistically significant change. Quadrafoil (Z440+Z441) showed a negative effect change from 0.020 ± 0.034 µm to −0.010 ± 0.027 µm at 6 months. Secondary astigmatism (Z420+Z421) had no statistically significant difference.

In this study, total HOAs significantly increased from 0.231 ± 0.089 µm at baseline to 0.654 ± 0.219 µm at 6 months after the commencement of OK lens wear. By contrast, total aberrations including lower-order aberrations decreased from 3.165 ± 0.914 µm at baseline to 1.623 ± 0.707 µm at 6 months. These changes in aberrations provide some empirical corroboration for the theoretical basis of OK to improve vision in myopic patients. However, no aberrations measured in this study showed significant correlation with the amount of correction in refractive error following the use of OK lenses, except for the changes in SA (Z400) at 3 and 6 months. Hiraoka et al. reported that decentration of an OK lens was related to an increase in coma (Z31) and SA (Z400) as well as a reduction in contrast sensitivity [21]; however, the reduction in contrast sensitivity was the only factor related to decentration by multiple regression analysis. In our study, all subjects showed similar central bull’s eye patterns in topography after wearing OK lenses; thus, no significant difference could be found related to decentration.

Subjects had significant improvements in UCVA and myopic spherical equivalent with properly prescribed OK lenses, and showed increases in total HOAs and SA (Z400), with decreases in defocus (Z200) and 0° (Z220) astigmatism at 1 month. The changes were maintained at 6 months. Vertical coma (Z311) and quadrafoil (Z440+Z441) significantly increased in negative directions at 1 month, but decreased at 3 months. The changes observed in 45° astigmatism (Z221), horizontal coma (Z310), trefoil (Z331), and secondary astigmatism (Z420+Z421) were not statistically significant. Moreover, it was noteworthy that SA (Z400) demonstrated statistically significant positive correlations with the change in spherical equivalent at 3 and 6 months, which showed the feasibility of serially checked SAs to evaluate the anti-myopia effect of the orthokeratology lens in children. 

This study has several limitations. First, concerning measurement of wavefront and HOAs, the constants of Zernike polynomials vary with the size of the pupils, making pupil size an important factor [22,23]. However, in the present study, the subjects were children aged between 9 and 15 years, with relatively small-sized pupils. Aberrometry measurements were difficult to perform because of poor cooperation and the difficulty in dilating pupils on an outpatient basis. According to previous data, contrast sensitivity functions may decrease after OK lens wear [18,21], but we did not perform any test for measuring contrast sensitivity that represents one’s quality of vision and discomfort. To solve these limitations, further study with a larger cohort of subjects and follow-ups greater than 6 months including tests for contrast sensitivity are required.

## 5. Conclusions

After wearing properly prescribed OK lens, all subjects had significantly improved UCVA and decreased myopic spherical equivalent, with increased total HOAs and positive spherical aberration at 1 month, and the changes were maintained at 6 months. Serially checked spherical aberration could evaluate the anti-myopia effect of the OK lens in children.

## Figures and Tables

**Table 1:** Demographics of the study group.

Demographics	Data
No. of eyes (patients)	15 (10)
Mean age (years, range)	11.5 (9–15)
Sex (male: female)	2: 13
Laterality (right: left)	7: 8
UCVA (logMAR)	0.96 ± 0.12
BCVA (logMAR)	0.00 ± 0.00
Keratometric mean K (D, mean ± SD)	43.49 ± 1.03
Keratometric mean astigmatism (D, mean ± SD)	1.15 ± 0.46
Spherical equivalent * (D, mean ± SD)	−2.88 ± 0.68
Refractive astigmatism ^†^ (D, mean ± SD)	−0.45 ± 0.59
Pupil size ^†^ (mm, mean ± SD)	8.13 ± 0.66

D: diopters; SD: standard deviation; K: keratometric readings. * measured with automated keratometer (KR-8800, Topcon corp., Tokyo, Japan). ^†^ measured with the Zywave^®^ aberrometer (Bausch & Lomb Inc., Rochester, NY, USA).

**Table 2:** Changes in refractive, keratometric and topographic values after wearing Orthokeratology lens (mean ± SD).

Clinical Parameters	Baseline	1 Month (*p* *)	3 Months (*p* ^†^)	6 Months (*p* ^‡^)	*p* ^§^
UCVA (logMAR)	0.96 ± 0.12	0.02 ± 0.02 (≤0.001)	0.02 ± 0.03 (≤0.001)	0.01 ± 0.02 (≤0.001)	≤0.001
BCVA (logMAR)	0.00 ± 0.00	0.00 ± 0.00 (1.00)	0.00 ± 0.00 (1.00)	0.00 ± 0.00 (1.00)	1.00
Spherical equivalent ^||^ (D)	−2.88 ± 0.68	−0.67 ± 0.37 (≤0.001)	−0.56 ± 0.36 (≤0.001)	−0.59 ± 0.36 (≤0.001)	≤0.001
Refractive astigmatism (D)	−0.45 ± 0.59	−0.45 ± 0.50 (1.00)	−0.48 ± 0.38 (0.91)	−0.48 ± 0.36 (0.91)	0.99
Keratometric steep K (D)	44.07 ± 1.05	42.70 ± 1.64 (≤0.001)	42.48 ± 1.49 (≤0.001)	42.47 ± 1.50 (≤0.001)	≤0.001
Keratometric flat K (D)	42.92 ± 1.06	41.57 ± 1.26 (≤0.001)	41.32 ± 1.16 (≤0.001)	41.37 ± 1.25 (≤0.001)	≤0.001
Keratometric mean K (D)	43.49 ± 1.03	42.13 ± 1.42 (≤0.001)	41.90 ± 1.30 (≤0.001)	41.92 ± 1.34 (≤0.001)	≤0.001
Keratometric astigmatism (D)	1.15 ± 0.46	1.13 ± 0.65 (0.92)	1.16 ± 0.57 (0.93)	1.10 ± 0.71 (0.74)	0.91
Topographic Sim K steep K (D)	44.08 ± 1.06	42.09 ± 1.21 (≤0.001)	41.99 ± 1.37 (≤0.001)	42.00 ± 1.37 (≤0.001)	≤0.001
Topographic Sim K flat K (D)	42.91 ± 1.01	41.31 ± 1.27 (≤0.001)	41.09 ± 1.14 (≤0.001)	41.09 ± 1.17 (≤0.001)	≤0.001
Topographic Sim K mean K (D)	43.5 ± 1.01	41.70 ± 1.19 (≤0.001)	41.54 ± 1.24 (≤0.001)	41.54 ± 1.25 (≤0.001)	≤0.001
Topographic Sim K astigmatism (D)	1.16 ± 0.43	0.78 ± 0.71 (0.08)	0.90 ± 0.48 (0.02)	0.91 ± 0.47 (0.02)	0.10
Topographic 5 mm steep K (D)	44.19 ± 1.18	43.69 ± 1.26 (0.08)	43.45 ± 1.09 (0.004)	43.44 ± 1.07 (0.003)	0.007
Topographic 5 mm flat K (D)	42.47 ± 1.11	41.28 ± 1.04 (≤0.001)	41.21 ± 1.07 (≤0.001)	41.20 ± 1.05 (≤0.001)	≤0.001
Topographic 5 mm mean K (D)	43.33 ± 1.09	42.49 ± 1.07 (≤0.001)	42.33 ± 1.02 (≤0.001)	42.32 ± 1.01 (≤0.001)	≤0.001
Topographic 5 mm astigmatism (D)	1.72 ± 0.68	2.41 ± 0.88 (0.03)	2.24 ± 0.69 (0.09)	2.24 ± 0.65 (0.08)	0.05
Pupil size ^#^ (mm)	8.13 ± 0.66	8.12 ± 0.69 (1.00)	8.27 ± 0.60 (0.92)	8.34 ± 0.55 (0.79)	0.72

SD = Standard Deviation, UCVA = Uncorrected visual acuity, BCVA = Best corrected visual acuity, D = Diopters, K = Keratometric readings, Sim K = Simulated Keratometric readings. * *p* value of changes in value after 1 month measured by repeated measure ANOVA and compared with baseline value. ^†^ *p* value of changes in value after 3 months measured by repeated measure ANOVA and compared with baseline value. ^‡^ *p* value of changes in value after 6 months measured by repeated measure ANOVA and compared with baseline value. ^§^ *p* value of changes in value measured by repeated measure ANOVA with Greenhouse–Geisser correction. ^||^ measured with auto-refractometer (KR-8800, Topcon corp., Tokyo, Japan). ^#^ measured with the Zywave^®^ aberrometer (Bausch & Lomb, Inc., Rochester, NY, USA).

**Table 3:** Changes in aberration values (mean of root-mean-square ± SD, µm) after wearing Orthokeratology lenses.

Parameters	Baseline	1 Month (*p* *)	3 Months (*p* ^†^)	6 Months (*p* ^‡^)	*p* ^§^
Total HOAs	0.236 ± 0.088	0.595 ± 0.205 (≤0.001)	0.643 ± 0.211 (≤0.001)	0.654 ± 0.219 (≤0.001)	≤0.001
Total aberrations	3.059 ± 0.979	1.644 ± 0.624 (≤0.001)	1.598 ± 0.619 (≤0.001)	1.623 ± 0.707 (≤0.001)	≤0.001
Defocus (Z200)	−4.102 ± 1.534	−3.113 ± 1.532 (0.003)	−2.905 ± 1.427 (≤0.001)	−2.878 ± 1.419 (≤0.001)	≤0.001
0° astigmatism (Z220)	0.401 ± 0.536	0.321 ± 0.609 (0.35)	0.149 ± 0.616 (0.01)	0.151 ± 0.614 (0.03)	0.01
45° astigmatism (Z221)	−0.198 ± 0.416	−0.031 ± 0.342 (0.25)	0.033 ± 0.483 (0.20)	0.036 ± 0.496 (0.29)	0.16
Horizontal coma (Z310)	−0.033 ± 0.138	0.108 ± 0.363 (0.16)	0.073 ± 0.360 (0.35)	0.065 ± 0.364 (0.43)	0.35
Vertical coma (Z311)	−0.114 ± 0.173	−0.463 ± 0.351 (≤0.001)	−0.086 ± 0.244 (0.78)	−0.094 ± 0.265 (0.73)	0.01
Trefoil (Z331)	0.094 ± 0.120	0.055 ± 0.100 (0.24)	0.043 ± 0.071 (0.09)	0.047 ± 0.079 (0.16)	0.21
SA (Z400)	0.159 ± 0.111	0.608 ± 0.197 (≤0.001)	0.640 ± 0.731 (≤0.001)	0.667 ± 0.825 (0.03)	0.05
Secondary astigmatism (Z420+Z421)	−0.003 ± 0.049	0.041 ± 0.133 (0.33)	0.012 ± 0.104 (0.59)	0.005 ± 0.116 (0.83)	0.42
Quadrafoil (Z440+Z441)	0.031 ± 0.053	−0.016 ± 0.039 (0.005)	−0.006 ± 0.035 (0.10)	−0.010 ± 0.022 (0.01)	0.03

RMS: Root-Mean-Square; SD: Standard Deviation; HOA: Higher-Order Aberration; SA: Spherical Aberration. * p value of changes in value after 1 month measured by repeated measure ANOVA and compared with baseline value. ^†^ *p* value of changes in value after 3 months measured by repeated measure ANOVA and compared with baseline value. ^‡^ *p* value of changes in value after 6 months measured by repeated measure ANOVA and compared with baseline value. ^§^ *p* value of changes in value measured by repeated measure ANOVA with Greenhouse–Geisser correction.

## Data Availability

Data are available upon request from the authors.
